# The High Risk of Bivalve Farming in Coastal Areas With Heavy Metal Pollution and Antibiotic-Resistant Bacteria: A Chilean Perspective

**DOI:** 10.3389/fcimb.2022.867446

**Published:** 2022-04-07

**Authors:** Alequis Pavón, Diego Riquelme, Víctor Jaña, Cristian Iribarren, Camila Manzano, Carmen Lopez-Joven, Sebastián Reyes-Cerpa, Paola Navarrete, Leonardo Pavez, Katherine García

**Affiliations:** ^1^ Instituto de Ciencias Biomédicas, Facultad de Ciencias de la Salud, Universidad Autónoma de Chile, Santiago, Chile; ^2^ Núcleo de Investigaciones Aplicadas en Ciencias Veterinarias y Agronómicas (NIAVA), Universidad de Las Américas, Santiago, Chile; ^3^ Instituto de Medicina Preventiva Veterinaria, Facultad de Ciencias Veterinarias, Universidad Austral de Chile, Valdivia, Chile; ^4^ Centro de Genómica y Bioinformática, Facultad de Ciencias, Universidad Mayor, Santiago, Chile; ^5^ Escuela de Biotecnología, Facultad de Ciencias, Universidad Mayor, Santiago, Chile; ^6^ Institute of Nutrition and Food Technology (INTA), University of Chile, Santiago, Chile; ^7^ Carrera de Nutrición y Dietética, Universidad Autónoma de Chile, Santiago, Chile

**Keywords:** bivalve farming, anthropogenic pollution, antimicrobial resistance, heavy metal resistance, *Vibrio* spp

## Abstract

Anthropogenic pollution has a huge impact on the water quality of marine ecosystems. Heavy metals and antibiotics are anthropogenic stressors that have a major effect on the health of the marine organisms. Although heavy metals are also associate with volcanic eruptions, wind erosion or evaporation, most of them come from industrial and urban waste. Such contamination, coupled to the use and subsequent misuse of antimicrobials in aquatic environments, is an important stress factor capable of affecting the marine communities in the ecosystem. Bivalves are important ecological components of the oceanic environments and can bioaccumulate pollutants during their feeding through water filtration, acting as environmental sentinels. However, heavy metals and antibiotics pollution can affect several of their physiologic and immunological processes, including their microbiome. In fact, heavy metals and antibiotics have the potential to select resistance genes in bacteria, including those that are part of the microbiota of bivalves, such as *Vibrio* spp. Worryingly, antibiotic-resistant phenotypes have been shown to be more tolerant to heavy metals, and *vice versa*, which probably occurs through co- and cross-resistance pathways. In this regard, a crucial role of heavy metal resistance genes in the spread of mobile element-mediated antibiotic resistance has been suggested. Thus, it might be expected that antibiotic resistance of *Vibrio* spp. associated with bivalves would be higher in contaminated environments. In this review, we focused on co-occurrence of heavy metal and antibiotic resistance in *Vibrio* spp. In addition, we explore the Chilean situation with respect to the contaminants described above, focusing on the main bivalves-producing region for human consumption, considering bivalves as potential vehicles of antibiotic resistance genes to humans through the ingestion of contaminated seafood.

## Introduction

The 2030 Agenda for Sustainable Development acknowledges the importance of water quality and aims to influence countries’ future policies and strategies to make water pollution control a global priority. Human settlements, industries and agriculture are the major sources of water pollution. Globally, 80% of municipal wastewater is discharged untreated into water bodies, and industry is responsible for dumping millions of tons of heavy metals (HMs), solvents, toxic sludge, and other wastes each year. The resulting water pollution is a demonstrated risk to aquatic ecosystems and human health (Mateo-Sagasta et al., 2017; Häder et al., 2020).

Anthropogenic pollutants include stressors of the biotic components of the aquatic ecosystem, such as HMs and antibiotics (Pierce and Ward, 2018). The global warming have a direct impact on acidification, temperature, and salinity of seawater, exacerbating the stressors mentioned above (Sugie et al., 2020).

Marine organisms such as bivalve mollusks are directly and indirectly affected by seawater pollution, which could alter the important ecological functions they perform in marine ecosystems. As filter-feeders, bivalves remove suspended material from the water, so they can concentrate water pollutants, which also explains their use as environmental sentinels (Dame, 2013; Bighiu et al., 2019; Giacometti et al., 2021). Bivalves are organisms that are not only important in marine ecosystems; for example, since the 1970s, the “Mussel Watch” program has been using mussels of the genus *Mytilus* to monitor pollutants (Goldberg et al., 1983; Benaltabet et al., 2021). Moreover, the impact of pollution is not only related to bivalves as filter-feeding organisms. The acidification of marine waters because of pollutants and climate change is harmful to the survival of calcium-containing organisms because it affects the development of bivalve mollusk shells (Landrigan et al., 2020).

Coastal zones represent approximately 7% of the marine environment, but their role in food productivity is key, providing more than 50% of the food in ocean ecosystems (Häder et al., 2020). Bivalve mollusks are harvested from coasts and estuaries reaching about 14% of the total marine production (Romalde et al., 2014). In addition, the world production of marine bivalves from aquaculture is approximately 89% of total production, the rest associated with wild fisheries (Wijsman et al., 2019).

Chile is the tenth largest producer of aquaculture products in the world, with salmon and bivalve mollusks as the main sources of fishery products (FAO, 2020; Naylor et al., 2021). Furthermore, the country is the second leading world´s producer and the main exporter of mussels (FAO, 2021). In addition, Chile is the second-largest producer of Atlantic salmon in the world, and the Chilean salmon production accounts for 27% of the world production (Morera et al., 2021). Salmonid and mollusk farming areas are concentrated in the southern areas of the country, mainly in Los Lagos and Aysén Regions (FAO, 2020).

**Figure 1:**
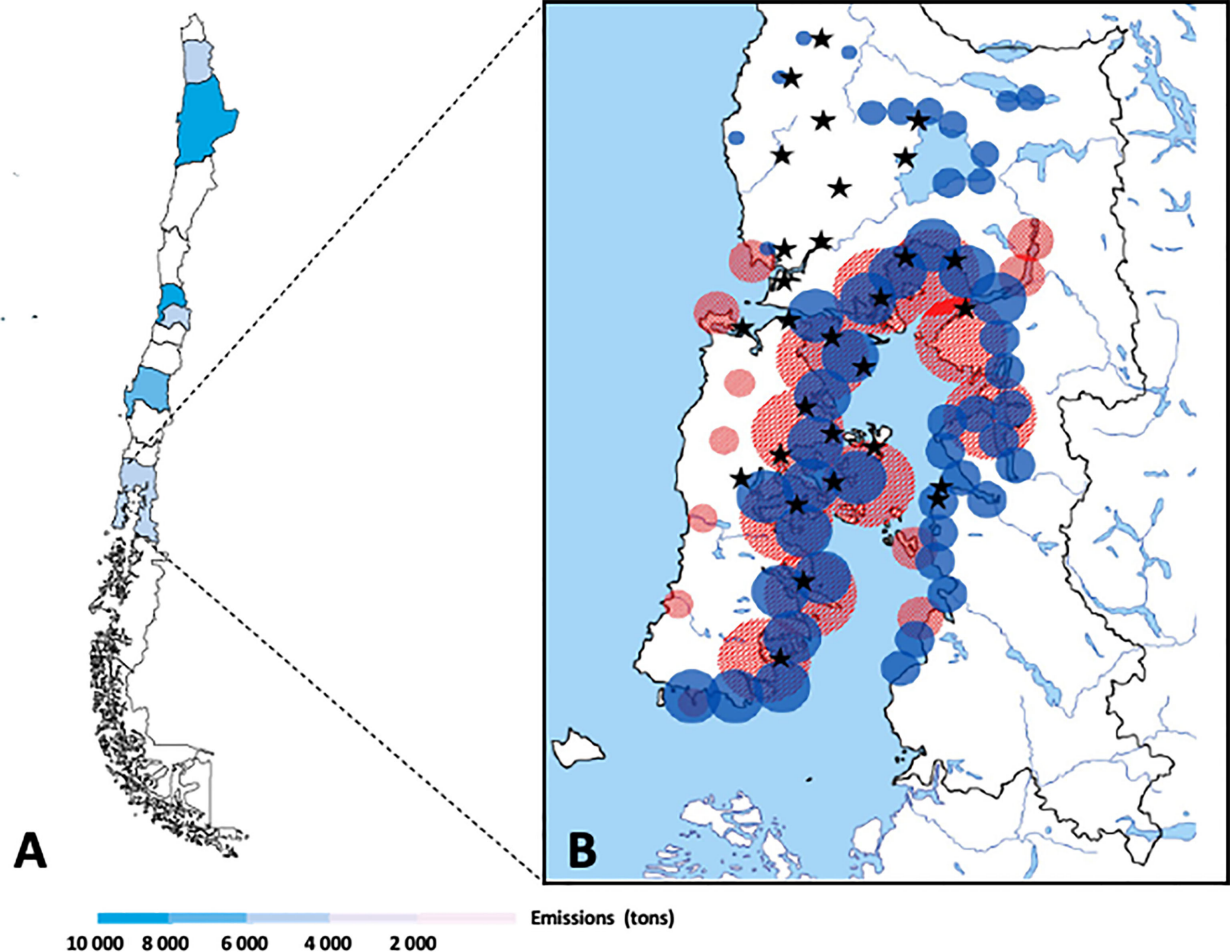
Heavy metal pollution in Chile. **(A)** Heavy metals discharged in each Chilean region. Metal emissions in tons (t) can be observed in horizontal line below the map figure. **(B)** Colocalization of fish culture centers (red circles), mussels culture centers (blue circles) and wastewater plants (black stars) in Los Lagos Region.

Chile’s geography, large coastal area on the Pacific Ocean, makes it very sensitive to seawater pollution and multiplies the impacts of climate change. Currently, in Chile there is an official order (Supreme Official Order 90/2000-Emission standard for the regulation of pollutants associated to the liquid waste discharges to sea, continental and superficial waters) that regulates the discharge of pollutants into marine and continental surface waters, establishing maximum permissible limits for the discharge of liquid wastes, thus avoiding the contamination of these bodies of water (SMA, 2000). However, compliance with emissions does not indicate that there is no discharge of pollutants because the cut-off value established in the official order is minimal. Today it is an obsolete and insufficient official order, which is being revised again by the Ministry of Environment to include the protection of estuaries, fjords and coastal wetlands and regulate parameters of chemical pollutants, such as HMs (Barra Ríos et al., 2021). In this regard, it is important to mention that Chile also does not have a legal framework that assigns emission reduction responsibilities or requires the implementation and reporting of measures to mitigate emissions and adapt to the impacts of climate change. However, a law with this objective was drafted in 2019 and is being debated in Congress (Madariaga Gómez de Cuenca, 2021). Although studies that determine the concentration of HMs in Chilean marine systems are scarce (Salamanca et al., 2000; Copaja et al., 2016; Espejo et al., 2019; Oyarzo-Miranda et al., 2020; Fierro et al., 2021), high concentrations have been found in some areas of the country. According to Chile’s pollutant emission and transfer registry (data can be obtained from https://datosretc.mma.gob.cl/group/emisiones-al-agua), the Los Lagos Region is among the five Chilean regions that contain the highest concentration of HMs and precisely this area is one of the main producers of bivalve mollusks such as mussels (**Figure 1A**). Several fish (red circles) and mussels (blue circles) culture centers are in this region, besides many wastewater plants (black stars), which together contribute to the high metal contamination observed in this area (**Figure 1B**).

This scenario of high concentrations of HMs is accompanied by the presence of antimicrobial resistant bacteria (ARB) in Chile’s aquatic ecosystems. However, in the last 20 years there are few publications focused on the co- and cross-resistance against antimicrobials and HMs (Domínguez et al., 2021).

The aim of this review is to analyze anthropogenic activities related to HMs and antibiotic contamination in areas producing bivalve mollusks, together with the impact on microorganisms, such as *Vibrio* spp., and their role in multiresistance phenomena, as well as to investigate how these contaminants could affect final consumers. In this regard, we hypothesize that in highly metal/antibiotics contaminated environments, microbial components of the bivalves could play a key role as carriers of resistant bacteria and their resistance genes, which could be transmitted to the human microbiota and affect human health.

## Anthropogenic Marine Ecosystems Pollution: An Approach to the Problem

In recent decades, there has been a steady and systematic growth of industries and population on a global scale, which has led to high anthropogenic pollution of the environment, including marine ecosystems. In fact, human activities are the main source of pollution originating from industrial and urban waste discharges that are often transported by water sources and atmospheric emissions that end up in the ocean (Chiang, 1989; Krumgalz, 1993; Valette-Silver, 1993; Bruland et al., 1994; Salamanca and Camaño, 1994). Eighty percent of ocean pollution comes from land-based sources, while discharges from marine shipping, offshore industrial operations, and waste disposal at sea account for the remaining 20%. Pollution is most severe along coasts, for example, in bays, harbors, and estuaries, where wastewater, industrial discharges, agricultural runoff, and river pollution are the main source. The coasts of the rapidly developing countries of the southern hemisphere have some of the worst ocean pollution in the world (Landrigan et al., 2020).

Rising sea surface temperatures and ocean pollution influence the abundance and geographic distribution of naturally occurring marine pathogens, such as *Vibrio parahaemolyticus and Vibrio vulnificus* (Baker-Austin et al., 2010). Therefore, the most likely consequences will be an increase in the frequency of *Vibrio*-associated diseases, as well as the spread of these infections to new, previously unaffected areas. The risk is especially high in countries where coastal development is intense, with dysfunctional sanitation systems and affected by sea level rise, coastal over-development and natural disasters (Landrigan et al., 2020).

Indeed, we know that ocean pollution is a complex phenomenon, involving a wide range of factors, including chemical and biological compounds, such as pesticides, petroleum derivatives, plastics, microplastics, wastewater, HMs, and antibiotics (Imran et al., 2019; Landrigan et al., 2020; Vaid et al., 2021; Zheng et al., 2021). However, in this review we will focus on the last two contaminants (HMs and antibiotics) mentioned above and how they may affect the environment of bivalve-producing areas, the microbial components of bivalves, emphasizing *Vibrio* spp., and as a result, the potential risk to human health from pathogenic species.

### Heavy Metals: A Resistance-Associated Stressor

Emissions of toxic metals to the environment began centuries ago with the onset of mining and smelting, continued to increase since the beginning of the Industrial Revolution, and have risen abruptly in the last two centuries (Landrigan et al., 2020). The classification of HMs includes any metallic chemical element with a density > 5 g/cm^3^, e.g., mercury (Hg), cadmium (Cd), arsenic (As), chromium (Cr), thallium (Tl), lead (Pb), silver (Ag), zinc (Zn), cooper (Cu), and iron (Fe). They exist naturally in the environment in the form of salts, minerals, and other types of compounds (Leong and Chang, 2020). A comprehensive assessment of HMs content in surface water bodies and the implications of toxic metals on aquatic life and human health was recently reviewed by Kumar and Collaborates (2019) and Okereafor and Collaborates (2020), respectively (Kumar et al., 2019; Okereafor et al., 2020).

Some HMs have important functions in biological systems. Through evolution, different organisms have acquired the ability to use some metals and HMs for essential biological functions (Nanda et al., 2019). Vanadium (V), manganese (Mn), cobalt (Co), nickel (Ni), molybdenum (Mo), boron (B), silicon (Si), selenium (Se), fluorine (F), iodine (I), tin (Sn), Cr, Fe, Cu, Zn, and As, are found in some living organisms. Generally, most of them are required as cofactors for enzymes, structural integrity or to provide a screen for electrostatic interactions in the aqueous phase. In this regard, they are essential in trace amounts but when they exceed their threshold values, they become toxic. Speciation of metals can also reduce or increase toxicity, for example, Cr (III) (trivalent) is known to be an essential trace element, while Cr (VI) (hexavalent) is highly toxic and the most toxic form of arsenic, As (III), is found in fractions of less than 20% of the total in marine systems. Moreover, organic forms of Hg are more toxic than inorganic and elemental Hg. Metal-protein complexes of metals such as Hg are intrinsically toxic and trace elements such as Zn also become toxic at high concentrations (Neff, 1997; Nies, 1999; Seiler and Berendonk, 2012; Fulke et al., 2020). The high concentration of other metals such as Ni, Cu and Co has been strongly associated with their toxicity (Richards et al., 2011). The presence of HMs in seawater has its origin naturally or anthropogenic way, but most of these substances come from industrial and urban waste. In fact, human activities are the main source of HMs pollution originating from industrial and urban waste discharges that are usually transported by water sources such as rivers and through atmospheric emissions (Chiang, 1989; Krumgalz, 1993; Valette-Silver, 1993; Bruland et al., 1994; Salamanca and Camaño, 1994). In addition, HMs are not biodegradable and tend to persist in nature and living organisms, resulting in accumulation that pollutes the environment and affects the food chain causing serious damage to health. The toxicity in the environment depends to a large extent on environmental conditions, as these influence the valence of the metal ions and thus their bioavailability (Suvarapu and Baek, 2017). There is a growing understanding of the anthropogenic impact of metal contamination on the microbial community of aquatic ecosystems (Ozbay et al., 2017; Yao et al., 2017; Igiri et al., 2018; Gillard et al., 2019; Rajeev et al., 2021; Liu et al., 2021).

The resistance and resilience of microorganisms to HMs in the ecosystem depends on many factors. For example, the intrinsic detoxification systems of microorganisms largely determine their survival in a contaminated environment. In addition, it is not excluded that other (a)biotic factors influence the toxicity of HMs and the fitness of microorganisms (Hao et al., 2021). Detailed information about the habitat and taxonomic distribution of HMs resistant bacteria isolated from polluted environments was discussed in a recent review (Hao et al., 2021).

The mechanisms by which bacteria develop resistance to metal toxicity can occur through different pathways, such as extracellular barrier, intracellular/extracellular sequestration, efflux, or reduction of metals ions (Igiri et al., 2018; Tarekegn et al., 2020) or by transference of resistance genes (Sterritt and Lester, 1980; Nanda et al., 2019). Yap and Collaborators (2007) mentioned that there is also a strong correlation between uptake and accumulation of trace metals in filter-feeders whereas they accumulate metal concentrations in water (Yap et al., 2007). As a result, aquatic living organisms become dangerous and threatening organisms to human being in terms of food (Yap et al., 2007). Therefore, ecosystems contaminated with HMs could exert a selective pressure that favors the development of resistant microorganisms. In this sense, the filtering characteristic of bivalves could multiply the possibilities of the appearance of bacteria resistant to HMs when bivalves, such as mussels, are cultivated in ecosystems contaminated with HMs. The consumption of these marine products would pose a latent risk to humans.

### Antibiotics: The Associated Risk of Resistance That Threatens Everyone

Antibiotics are substances able to inhibit the growth or killing bacteria. According to World Health Organization (WHO), antimicrobial resistance occurs due to genetic changes over time, and resistance genes can spread among living organisms (WHO, 2020). ARB cause more than 100.000 deaths per years in China, 58.000 in India, 35.000 in the United States and 33.000 in the European Economic Area and these numbers should increase because of rapid socioeconomic development along with population growth (Reverter et al., 2020; Domínguez et al., 2021). In Latin America, multi-drug resistant organisms are the leading cause of hospital acquired infections (Domínguez et al., 2021). Moreover, infections produced by resistant bacteria are associated with increased mortality, morbidity, and greater numbers of complications, prolonged hospitalizations, and more expensive treatments. This phenomenon is especially recurrent in countries that use high amounts of antimicrobials in animal production industries and for veterinary care (Millanao et al., 2018). According to surveillance data from the Latin American Network for Antimicrobial Resistance Surveillance (ReLAVRA) an increased trend has been observed in carbapenem resistant bacteria since 2014 (Domínguez et al., 2021). Acquired resistance in a pathogen occurs through a stepwise evolution from a chromosomal and immobile ARGs, In addition, ARGs genes existed in the environment before antimicrobials were discovered and used as therapeutic agents, however, their use and subsequent misuse induced natural selection of resistant bacteria (Lupo et al., 2012).

Bacterial pathogens are the main pathway for the spread of ARGs from “hot spots” of potential resistance development (Ravi et al., 2014). Even resistance gene transfer events can be stimulated by antibiotics themselves (Allen, 2017). The rate of HGT in human-associated bacteria is up to 25-fold higher than HGT in non-human-associated bacteria (Smillie et al., 2011; Ravi et al., 2014).

In bacteria, the most common HGT mechanisms are conjugation, transformation, and transduction. The spread of antibiotic resistance (AR) determinants among the bacterial community is mainly attributed to the rapid HGT of genetic elements, such as integrons, plasmids, and transposons (Zheng et al., 2021). The plasmid transfer between bacteria could occur within an hour, despite suboptimal conditions and the absence of selective pressure (Ravi et al., 2014).

Novel resistance genes that are so far not clinically important may arise from the vast reservoirs of environmental and commensal bacteria due to selective pressure. Compared to anthropogenically selected resistance genes, these novel genes are usually not found in mobile genetic elements (MGE) such as integrons, transposons and plasmids, so they must be selected in several steps in MGE before they arrive a pathogenic bacterium (Stokes and Gillings, 2011; Allen, 2017). For example, reviewing the literature we found a study demonstrating the relationship between ARGs in marine bacteria and human uropathogenic *Escherichia coli*, in an intensive aquaculture region (Tomova et al., 2015). The authors identified ARGs in marine bacteria isolated from Chilean aquaculture and non-aquaculture sites that endowed them with resistance to tetracycline, florfenicol and quinolones. They demonstrated that plasmid-mediated quinolone resistance genes were present more frequently in uropathogenic *E. coli* isolates from a coastal site bordering Chilean aquaculture than in uropathogenic *E. coli* isolates from an urban non-aquaculture site in the United States (Tomova et al., 2015). In addition, some Chilean uropathogenic *E. coli* had plasmid-mediated quinolone resistance genes whose sequences were identical to those of local Chilean marine bacteria, suggesting linkage of these marine and terrestrial bacterial populations through uni- or bidirectional HGT mediated gene flow (Tomova et al., 2015). The above is an example of the public health risk associated with AR when human settlements and industries dedicated to the intensive farming of animals coexist, such as aquaculture, and the prevailing need to maintain good veterinary practices that favor the hygiene and well-being of animals, but also decrease the impact of the spread of ARGs.

Moreover, *Aeromonas* spp. members could easily develop single or multiple AR phenotypes (Janda and Abbott, 2010) playing an important role in the dissemination of AR in aquatic environments as indicated by Figueira and Collaborates (2011) (Figueira et al., 2011). Another example is the *blaCTX-M* genes, which is currently the most prevalent cause of extended spectrum β-lactamases (ESBL) in *Enterobacteriaceae* worldwide and a major cause of clinical treatment problems (Hawkey and Jones, 2009). The most probable origin of these genes has been identified in the chromosomal DNA of several environmental species of *Kluyvera* spp., where it is believed to have spread with great success to different bacterial species (Cantón and Coque, 2006). Furthermore, in *Shewanella algae* was found the origin of plasmid-encoded *qnrA* genes, which confers resistance to quinolones (Poirel et al., 2005). Likewise, OXA-48 genes, which possess remarkable carbapenem-hydrolyzing activity, are increasingly prevalent in *Enterobacteriaceae* species worldwide and originated in the chromosomes of *Shewanella* spp. from the aquatic environment (Poirel et al., 2012). Some species of the *Vibrionaceae* family are part of the microbiota of mussels and they could be the reservoir of other plasmid-encoded qnrA genes (Poirel et al., 2005), which have spread worldwide in several species of *Enterobacteriaceae*. Worryingly, the antimicrobial resistance emerged and evolved in *Vibrio* species over the last decades has been matter of concern, since multiresistance of fish pathogenic species has caused important economic loses to the aquaculture industry. Even worse, AR is induced in the surrounding bacteria in the column water, sediment, and fish-associated bacterial strains, and the use of antibiotics in aquaculture also impacts the frequencies of resistance in human pathogens (Pepi and Focardi, 2021). In fact, human pathogenic species of *Vibrio* have increased resistance to several clinical antibiotics of common use during last years (Mohamad et al., 2019).

### Wastewaters: Convergence of Marine Pollutants

Municipal and industrial wastewater treatment plants (MIWTP) are important point sources of nutrients and organic material that alter biogeochemical processes in receiving aquatic ecosystems (Lofton et al., 2007), but they also contain contaminants that have the potential or capacity to alter ecosystems throughout the trophic level. Contaminations such as HMs and antibiotics are a great interest because they could cause changes in the function, composition and resistome of the microbial communities present in sediments and marine estuaries (Wakelin et al., 2008). Wastewater treatment plants are considered one of the main sources of AR and it is estimated that between 75%-90% of antibiotics are poorly absorbed by humans or animal hosts and excreted, unaltered, in feces or urine (Uyaguari-Díaz et al., 2018). Despite all treatments of wastewaters, they are finally released to estuaries, rivers and oceans containing several ARGs. Thus, the discharge of antibiotics and their metabolites into the environment is widespread; “hot spots” of contamination include wastewater discharges from hospital, healthcare facilities, community wastewater treatment plants, pharmaceutical industry, and confined animal feeding operations (Pruden et al., 2006; Berendonk et al., 2015). Some antibiotics can degrade rapidly, while others accumulate in soil or sediments and persist into the environment for longer (Shi et al., 2014). Surface water remains the main vehicle for the dissemination of antibiotic resistant bacteria (ARB), antibiotic residues, and ARGs in the environment (Heberer, 2002; Uyaguari-Díaz et al., 2018). Worryingly, the interaction between environmental and clinical microorganisms could select multidrug-resistant bacteria, severely affecting environments, changing biodiversity, and modifying evolutionary pathways in favor of resistant ones (Eduardo-Correia et al., 2019). Furthermore, HMs concentrations detected in wastewater treatment plants are generally two or four orders of magnitude higher than antibiotics levels (Seiler and Berendonk, 2012). These metals, as the antibiotics, are not subjected to rapid degradation, ensuring a maintenance of selection for HMs resistance (Stepanauskas et al., 2005).

We suggest that the discharge of HMs, together with antibiotics from agriculture and ecosystems linked to animal production into the seawater environment, can cause a combined selection and co-selection effect toward ARB development.

## Antibiotics and Heavy Metal: Resistance Co-Selection Factors

Indirect selection for AR by HMs through co-selection has been of concern since the 1970’s (Koditschek and Guyre, 1974). This indirect process occurs by physiological (cross-resistance) and genetic (co-resistance) coupling of antibiotic and HMs resistance mechanisms. Cross-resistance describes mechanisms that provide tolerance to more than one antimicrobial agent, such as antibiotics and HMs.

On the other hand, in co-resistance, two or more resistance genes are physically linked and the genes responsible for two or more resistances are located together in a MGE, as plasmids, integrons or transposons, and could potentially propagate to other bacterial species through HGTs (Chapman, 2003; Seiler and Berendonk, 2012; Pal et al., 2017; Verma et al., 2019; Zou et al., 2021). Well-characterized mechanisms of cross-resistance and co-selection can be detailed reviewed in Baker-Austin and Collaborates (2006); Seiler and Berendonk (2012) and Imran and Collaborates (2019) (Baker-Austin et al., 2006; Seiler and Berendonk, 2012; Imran et al., 2019).

Co-selection of HMs resistance genes (HMRGs) and ARGs have been reported in agriculture (Hu et al., 2016), livestock (Zhu et al., 2013), sediment (Wright, 2010), mining (Zou et al., 2021), pond sediments (a microbial paleontology approach) (Dickinson et al., 2019) and wastewater treatment system (Di Cesare et al., 2016a; Di Cesare et al., 2016b). However, MIWTPs are particularly recognized as a “hot spot” of transfer between environmental and pathogenic bacteria (Guo et al., 2017; Manaia et al., 2018). The simultaneous presence of chemicals stress (HMs and/or antibiotics at sublethal concentrations), resistant bacteria, and resistance genes can favor the selection of multidrug-resistant bacteria and the potentiation of resistances in the environment (Shendure and Ji, 2008; Di Cesare et al., 2016a; Shen et al., 2021; Silva et al., 2021).

In the presence of stress, the selection of the corresponding resistance gene promotes the persistence of other resistance genes, even without a direct impact of their specific stressors (Chapman, 2003; Di Cesare et al., 2016a). HMs have been suggested to enhance selection for AR in the environment and *vice versa* through co-resistance, cross-resistance or co-regulation of resistance pathways (Matyar et al., 2008; Zhang et al., 2021). In this regard, experimental evidence demonstrated a relationship between HMRGs and ARGs acquisition, both of which disseminated through the MGE (Szczepanowski et al., 2005; Tennstedt et al., 2005; Baker-Austin et al., 2006; Szczepanowski et al., 2007; Graham et al., 2011; Knapp et al., 2011; Di Cesare et al., 2016a); these mechanisms were extensive review by Seiler and Berendonk, 2012 (Seiler and Berendonk, 2012). Such elements, referred to as integrative and conjugative elements, are self-transmissible chromosomal MGE that encode a wide variety of genetic information: a characteristic set of core genes (for excision, circularization, conjugative transfer, and site-specific integration) and cargo genes, which confer a wide range of phenotypes to their hosts, including antibiotic and HMs resistance (Wozniak and Waldor, 2010; Durrant et al., 2020).

The co-selection mechanism is highly favored when diverse resistance genes are located on the same MGE, for example: an integron, a plasmid, a genomic island, a phage, or a transposon, facilitating the lateral transfer in the worldwide problem of resistance (Chapman, 2003; Gillings et al., 2008; Di Cesare et al., 2016a; Uyaguari-Díaz et al., 2018). The genomic plasticity of MGE has contributed to the fitness quotient and robustness of bacteria to survive in different environments. In addition, plasmid-borne or transposon-enclosed integrons disseminate the resistance gene and play an important role in the development and spread of superbugs (Sultan et al., 2018). Integrons provide bacteria with rapid adaptation under strong selection pressure, so they are considered the main agents of bacterial evolution due to their role in the ARGs spread, development of multidrug resistance and potential to add genetic structures in bacterial genomes (Gillings et al., 2008; Uyaguari-Díaz et al., 2018).

There are examples in the literature describing the role of HMs in increasing AR of microorganisms in aquatic ecosystems. In a recent study, Wang and Collaborates (2021) confirmed that the selective pressure of HMs contributed to the increase in ampicillin-resistant opportunistic pathogens (*Pseudomonas monteilii*, *Aeromonas hydrophila*, *Acinetobacter baumannii*, and *Staphylococcus epidermidis*) in the Xiangjiang River, China. In addition, a microcosm experiment showed that the HMs (Cu^2+^ and Zn^2+^) raised the abundance of β-lactam resistance genes carried by opportunistic pathogenic bacteria and the horizontal transfer of plasmids in pathogenic bacteria (Wang et al., 2021).

Other HMs, such as Zn, Cd, and Hg have been associated with methicillin resistance on *Staphylococcus aureus* chromosomes (Ito et al., 2001; Cavaco et al., 2010). We have previously mentioned that HMs can persist in the natural environment for long periods of time; therefore, according to some authors, their contribution to the maintenance and spread of AR factors may be more than we expected (Baker-Austin et al., 2006; Ji et al., 2012). However, more studies are needed in long-term HMs and antibiotic contamination in relation with evolutionary bacterial communities.

We have already mentioned that integrons play a key role in ARGs propagation and they are considered the main agents of bacterial evolution. The most studied integrons are the resistance integrons, in which the cassettes are antibiotic resistance determinants (Xu et al., 2007). Two classes of integrons (IV and V) have been identified in *Vibrio* spp, with some species that are part of the microbiota of mussels, and both were associated with the development of resistance to the antibiotic trimethoprim (Ravi et al., 2014). In addition, the integrons in *Hydrogenophaga* spp., *Imtechium* spp., and *Aquabacterium* spp carried a gene cassette whose open reading frame was homologous to a hypothetical protein (VP1784) from *Vibrio parahaemolyticus*. This cassette was also present in the previously described class 1 integron from *Acidovorax* sp, a specie that was isolated from a wastewater treatment plant and belongs to a genus with clinically relevant species (Gillings et al., 2008; Wisplinghoff, 2017).

Importantly, in approximately 70 years, the integron has accelerated resistance mechanisms to the point of rendering antibiotic treatment ineffective, which is a major health concern (WHO, 2012). The hypothesis that ancestral integrons were not associated with ARGs compared to current integrons reinforces the fact of antibiotics overuse and misuse (Uyaguari-Díaz et al., 2018).

## Impact of Seawater Pollution in Bivalves and *Vibrio* spp.

Bivalves have different physiological characteristics, such as stress resistance, sessile behavior, tolerance to salinity changes and the ability to accumulate contaminants at levels higher than those found in seawater, making them very useful for monitoring levels and trends of classical and emerging contaminants (Rodil et al., 2019). They are an important filtering organism and have been used as bioindicators in environmental monitoring programs. For example, since 1986 the National Oceanic and Atmospheric Administration (NOAA) National Mussel Watch Program has conducted annual sampling of the bivalve *Mytilus edulis*, or similar species, along the U.S. coast to assess the status and long-term trends of approximately 140 contaminant analytes (Bricker et al., 2014).

We previously discussed the role of HMs in the environment as a stress factor that may favor the potential development of resistance in marine bacteria and its risk to human health. In addition to this role, HMs also affect the development of bivalves. During feeding, bivalves in ecosystems contaminated with HMs can bioaccumulate them through water filtration (Wang and Lu, 2017; Max Blanc et al., 2019; Yuan et al., 2020). The incorporation of these metal compounds also depends on the physical conditions of the metal and the physicochemical factors of the environment (Wang and Rainbow, 2005; Tapia et al., 2010; Cardwell et al., 2013). Several investigations have shown that HMs can affect physiological processes in bivalves (Mandich, 2018). There is evidence that HMs cause damage to the gills and hepatopancreas of green mussels (*Perna viridis*) and, being teratogenic, interfere with their reproduction (Ferraro et al., 2006; Riani et al., 2018). Other studies showed that Hg (2 x 10^-4^ M) caused high hemocyte mortality in *Crassostrea gigas*, while Cd^2+^ exposure (10-100 μmol L^-1^) was associated with high levels of apoptosis in *Crassostrea virginica*. Moreover, mussels exposed to Cu (20 μg L^-1^) and Hg (20 μg L^-1^) affected several immune parameters, including phagocytosis and *Mytilus galloprovincialis* decreased its lysosomal membrane stability of hemocyte in response to Cr (0.1, 1, 10 and 100 μM) (Renault, 2015).

Unfortunately, coastal marine areas and estuaries, including bivalve mollusk farming areas, are prone to high levels of HMs contamination (Stewart et al., 2021). A recent study performed in bivalves marketed in coastal cities of China showed that, due to their water filtering capacity, the mean concentration of HMs in bivalve tissues decreased in the following order Zn (5.29–35.74, mean: 12.37 ± 5.58 mg/kg) > Cu (0.74–4.93, mean: 1.72 ± 0.72 mg/kg) > As (0.61–3.95, mean: 1.50 ± 0.81 mg/kg) > Cd (0.02–0.35, mean: 0.12 ± 0.07 mg/kg.) > Cr (0.06–1.09, mean: 0.30 ± 0.15 mg/kg) >Pb (0.03–0.48, mean: 0.19 ± 0.10 mg/kg) > Hg (0.001–0.029, mean 0.008 ± 0.006 mg/kg) (Dame, 2013; Qin et al., 2021).

However, it is important to note that despite several studies on the accumulation of HMs in mussels (Chan, 1989; Naimo, 1995; Yap et al., 2003; Fung et al., 2004; Zuykov et al., 2013; Liu and Wang, 2016), their retention and depuration dynamics remain of increasing interest (Stewart et al., 2021) because of the physiological consequences and possible implications related to human consumption.

On the other hand, the acidification of seawaters environments, resulting from anthropogenic pollution and increased CO_2_ concentrations, has a direct effect on the increased toxicity of certain HMs, such as Cu, in bivalves (Pascal et al., 2010; Yang et al., 2019; Landrigan et al., 2020). Moreover, in certain estuaries, the mean free ionic form of Cu^2+^ could increase 115% by the year 2100, due of a possible decrease of ocean pH to 7.7 (Landrigan et al., 2020). An example of how Cu affected *Mytilus californianus* larvae and adults was demonstrated by Hall et al. (2020). This metal to 25 μg L^-1^ caused developmental and neurological malfunction, specifically a drastic structural malformation, erratic swimming behavior of larvae and failure in shell development (Hall et al., 2020).

Bivalve mollusks constitute habitats for bacteria of the *Vibrionaceae* family in the marine environment. In this regard, *Proteobacteria* has been previously described as a predominant phylum in many HMs polluted environments, with a variety of HMRGs and strong capability of adaptation and tolerance. In addition, species of Gram-negative γ-proteobacteria in the genus *Vibrio* spp. make up a significant fraction of the culturable heterotrophic bacteria of oceans and estuaries (Hao et al., 2021).

On the other hand, the microbiota of bivalves is fundamental to their homeostasis (Lohrmann et al., 2019). By filtering food particles from seawater, bivalves accumulate exogenous bacteria, often transiently (Froelich and Oliver, 2013). This filter feeding process also allows the accumulation of pathogenic organisms in the bivalves, thus acting as passive carriers of human pathogens (Lopez-Joven et al., 2011; Leite et al., 2017; Bighiu et al., 2019; Loo et al., 2020; Giacometti et al., 2021). We have already mentioned that in the seawater environment, bivalves constitute habitats for components of the *Vibrionaceae* family, and some regular components of bivalve microbiota correspond to *Vibrio* spp. (Pruzzo et al., 2005). In fact, *Vibrio* spp. are components of the microbiota of oysters and mussels, concentrating these bacteria in their tissues and hemolymph (Le Roux et al., 2016; Destoumieux-Garzón et al., 2020). The pathogenicity of *Vibrio* spp. is related to temperature, a climatic parameter greatly affected by contamination. The increase in sea surface temperature favors a higher rate of transmission, proliferation, and changes in the regulation of several virulence factors involved in motility, host degradation, secretion, and antimicrobial resistance of *Vibrio* spp. (Warner and Oliver, 2008; Kimes et al., 2012; Ceccarelli et al., 2013; Mok et al., 2019), increasing host susceptibility by weakening the bivalves immune system (Harvell et al., 2009). The high density in current bivalve culture systems stresses these organisms, which also leads to decreased immune functions and thus lower resistance to disease (Lohrmann et al., 2019). In this sense, the presence of pathogenic species for bivalve mollusks directly affects the mussel farming. Nevertheless, other components of the *Vibrionaceae* family can infect humans, so the possibility of containing high loads of pathogenic bacteria is a risk factor for bivalve consumers’ health.

Clinically, the most important human pathogens of *Vibrio* spp. are *V. cholerae*, *V. vulnificus* and *V. parahaemolyticus* (Thompson et al., 2004; Pérez-Reytor et al., 2018). In particular, *Vibrio parahaemolyticus* comprises many strains that inhabit the Chilean coastal sea. However, a pandemic strain was first observed in Chile in 1998 (Antofagasta), when it produced a large outbreak. Six years later, diarrhea outbreaks related to seafood consumption began in Los Lagos Region. Until 2009 the pandemic strain was a relatively stable bacterial subpopulation of the diverse *V. parahaemolyticus* population present in shellfish (Garcia et al., 2009), but in 2011 the pandemic strain disappeared in the region, completing a rise and fall cycle previously observed in other countries (Garcia et al., 2013). Nonetheless, other post-pandemic *V. parahaemolyticus* strains have been associated with clinical cases, including strains lacking the major toxins of this bacterial species (Castillo et al., 2018).

Although most infections associated with human pathogenic *Vibrio* spp. are self-limiting, severe cases of the disease require antibiotics for treatment. However, several reports have warned about the increase in AR of environmental *Vibrio* spp. that is associated with the use of antibiotics for the treatment of vibriosis (Loo et al., 2020).

The occurrence of AMR in *Vibrio* has increased worldwide and the efficacy of clinically important antibiotics has declined, emerging as a global threat to public health (Dutta et al., 2021).

Resistance to antibiotic ampicillin, chloramphenicol, cephalosporins and tetracycline has been detected in *Vibrio* spp. (Lloyd et al., 2018; Loo et al., 2020), but recently it has been reported that *V. cholerae* can avoid the effects of almost all antibiotics used for the treatment of cholera and others bacterial infectious diseases (Das et al., 2020).

In addition, antibiotic-resistant phenotypes of *Vibrio* spp. are more tolerant to HMs, and *vice versa*, which probably occurs through co-resistance pathways (Matyar, 2012; He et al., 2016; Xu et al., 2019). Resistance of *V. cholerae* to antimicrobials and HMs has been previously reported (Song et al., 2013; Bhuyan et al., 2016; Baron et al., 2017; Sulca et al., 2018; Xu et al., 2019). On the other hand, a very important aspect to consider is that HMs tolerance was prevalent in the *V. parahaemolyticus* strains with more than two AR phenotypes (He et al., 2016; Kang et al., 2018; Jiang et al., 2020).

Despite the ecological importance and the risk to human health associated with resistance co-selection, (Vaz-Moreira et al., 2014; Hu and Chen, 2016; Lloyd et al., 2018; Squadrone, 2020; Silva et al., 2021) less attention has so far been focused on the simultaneous occurrence of HMRGs and ARGs in *Vibrio* spp. Due to their ability to relocate between host genomes, *Vibrio-*MGE could play a vital role by acting as vehicles for the acquisition of resistance gene and their successive propagation. In this sense, bivalves are a potential vehicle of antibiotic‐resistant *Vibrio* spp., where resistance genes could be transmitted to humans through ingestion of marine food (Lloyd et al., 2019; Loo et al., 2020).

## Chile: Antibiotics and Heavy Metals as Marine Pollutants in Los Lagos Region

The consumption of fish and seafood will increase 27% by 2030, mainly due to the aquaculture sector, which will grow by 62%. The shift in the human diet towards increased consumption of fish and seafood is suggested to be a solution to the need for protein that supports human and environmental health (FAO, 2020; Reverter et al., 2020). However, aquaculture has high costs associated with its development. Chilean aquaculture grew 158-fold during the last three decades (Poblete et al., 2019). Currently, Chile is the world´s tenth largest producer of aquaculture products in the world, with salmonids (*Salmo salar*, *Oncorhynchus kisutch* and *Oncorhynchus mykiss*) and mollusks bivalves (*Mytilus chilensis*) being the main products (FAO, 2020; Naylor et al., 2021). The country is the second leading world´s producer and the main exporter of mussels (FAO, 2021), and second-largest producer of *Salmo salar* in the world (Morera et al., 2021).

**Figure 2:**
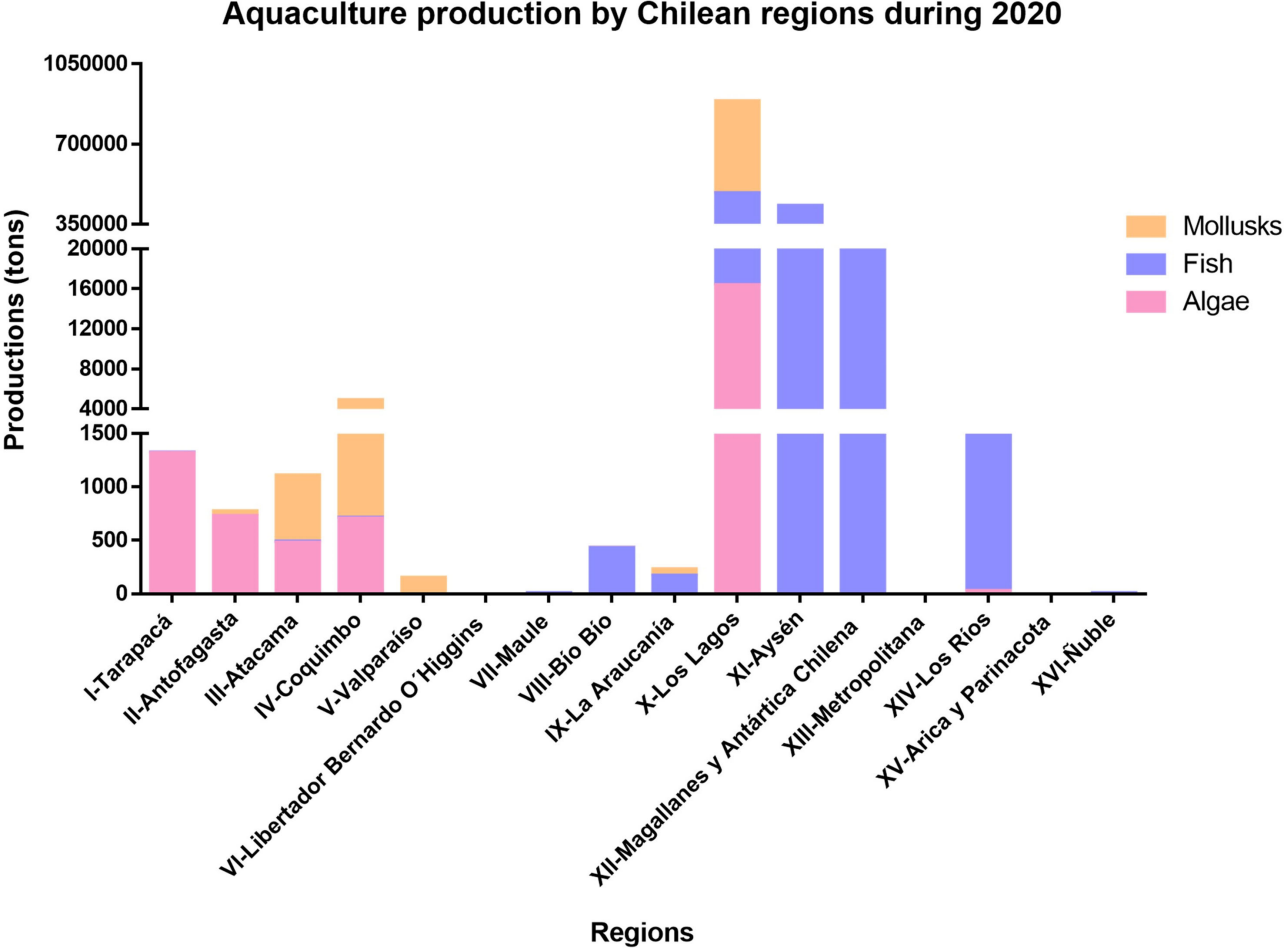
Aquaculture production in Chile. Production of mollusks, fish, and algae (expressed in tons, y-axis) in each Chilean region during 2020.

**Figure 3:**
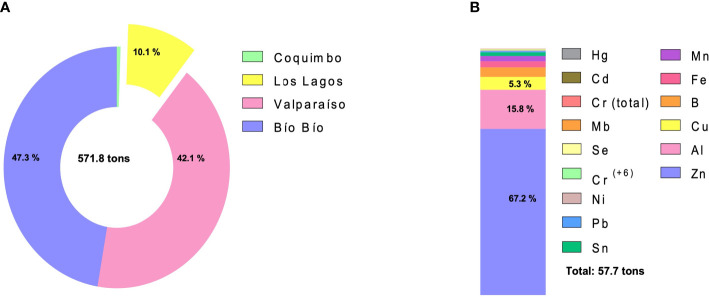
Heavy metals discharged in Chilean coasts during 2019. **(A)** Percentage of heavy metals spill (expressed in tons) in major regions of mussel’s cultivation during 2019. **(B)** Percentage of each heavy metal spilled (in tons) in Los Lagos Region during 2019.

Cultivation areas are concentrated in the south of the country, mainly in Los Lagos and Aysén Regions (FAO, 2020) (**Figures 1B**, **2**). However, Los Lagos Region is among the five Chilean regions with the highest concentration of HMs (**Figure 1A**) and it is also the third largest mussel cultivation region (**Figure 3A**). The HMs mostly discharged in Los Lagos Region during 2019 were Zn, Al, and Cu (**Figure 3B**). In this regard, Nguen and Collaborates (2019) showed that across diverse environmental reservoirs (water, wastewater and soil), Zn and Cd were the most observed HMs associated with ARGs (Nguyen et al., 2019).

**Figure 4:**
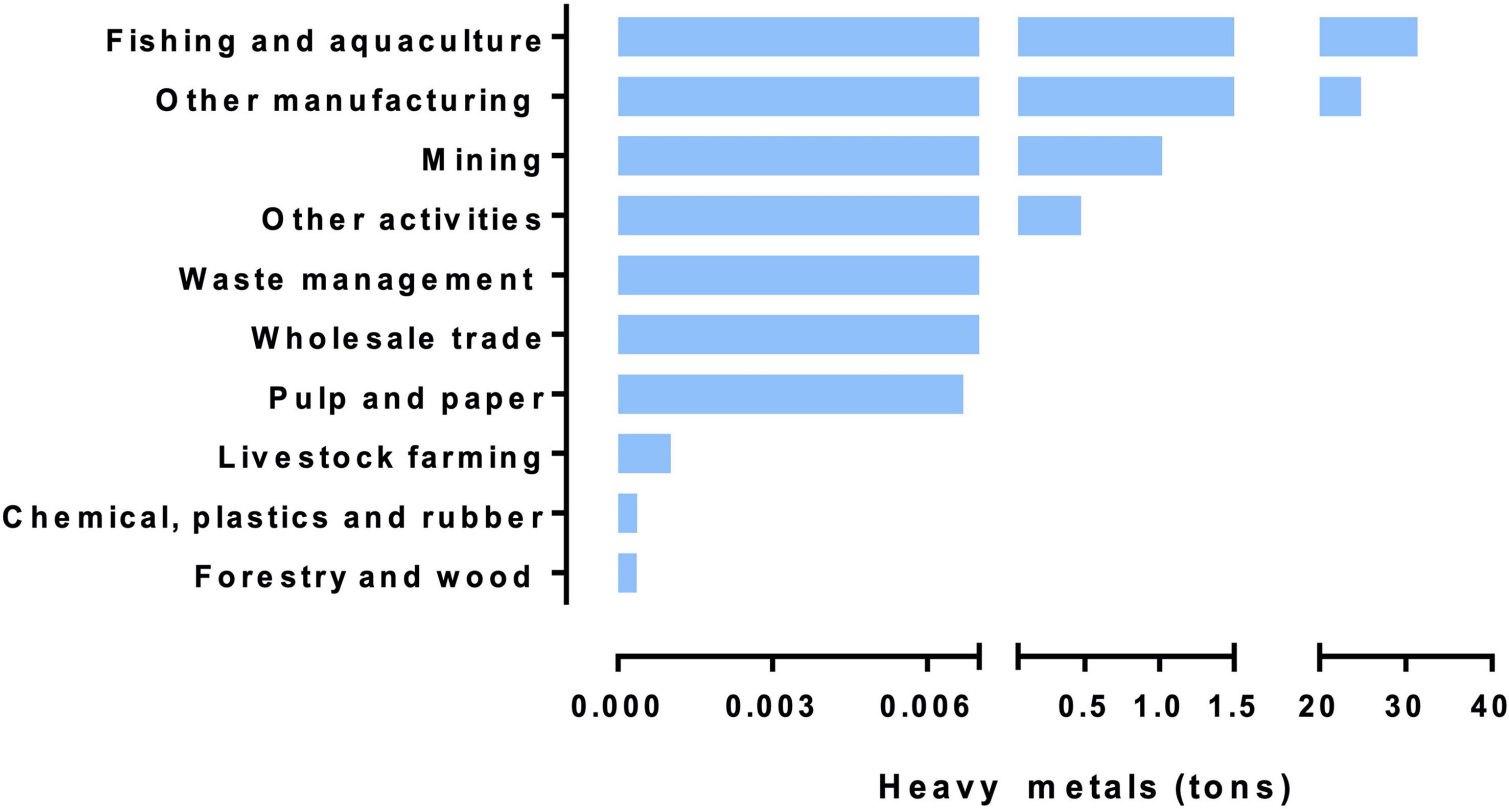
Contribution of heavy metals discharged by diverse industries in Los Lagos Region during 2019. Heavy metals are expressed in tons (x-axis). Different industries are listed in y-axis. Data source: http://datosretc.mma.gob.cl/dataset/emisiones-al-agua/resource/041eb3e7-87b0-4be3-a980-4c837b02e97f and https://snifa.sma.gob.cl/Estadisticas/Resultado/5.

**Figure 5:**
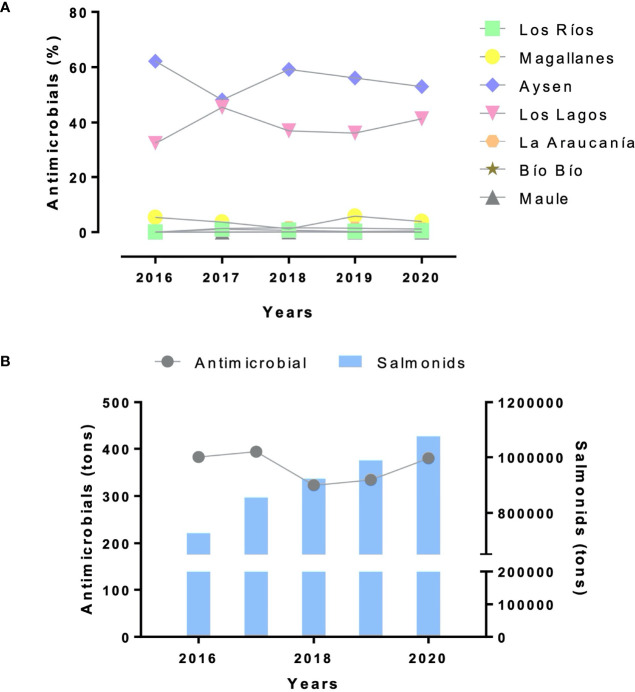
Antimicrobial use in Chilean regions. **(A)** Percentage of antimicrobials (y-axis) used in major regions of aquaculture’s production and other regions during 2016-2020 (x-axis). **(B)** Comparison of antimicrobial use (in tons) and salmonid production (in tons) occurred in Los Lagos Region during the same period (2016-2020, x-axis).

Notably, according to Chilean National System of Environmental Information (SNIFA) and Pollutant Emission and Transfer Registry of Chile (RETC) the aquaculture is the industry that mostly discharge metals into the waters in Los Lagos Region due to the production of balanced feed, which include minerals and fishmeal obtained from raw materials containing metals (Choi and Cech, 1998), furthermore, fertilizers applied to fish farms may contain heavy metals (Emenike et al., 2021). In addition to the high concentration of HMs, there are also several wastewater plants that discharge the treated water into the coasts of Los Lagos Region (**Figure 4**). Regarding MIWTP, Chilean environment legislation regulates the discharge of pollutants into marine waters. The main controls about secondary emissions to body waters is regulated for the Supreme Official Order 90/2000-Emission standard for the regulation of pollutants associated to the liquid waste discharges to sea, continental and superficial waters and Supreme Official Order 46/2002-Emission standard for liquid industrial waste (ILW) discharged to groundwater (SMA, 2000; SMA, 2002). Both supreme official orders only regulate physical, chemical, and bacteriological parameters, excluding the analysis of antibiotics and the identification of bacterial pathogens. Indeed, it is not possible to determine the quantity, concentration and type of antibiotic that are deposited in water sources from sanitary industries. In addition, the difficulty of controlling the flow of water discharged into the ocean, could potentially further increase the presence of antibiotics in coastal and ocean waters. Contributing to the perfect storm, the development of salmon farming in Chile has been accompanied by the misuse of antibiotics, with the highest percentages concentrated in Aysén and Los Lagos Regions (**Figure 5A**). The emergence of infectious diseases is currently the most serious problem facing aquaculture worldwide (FAO, 2020). In addition, the development of AR and its associated environmental impacts is a growing public concern that is challenging, for example, the growth of aquaculture (**Figure 5B**). Excessive amounts of antibiotics continue to be used in Chile, which plays an important role in the emerging public health crisis of AR. Resistant bacteria as well as antibiotic residues from salmon production are spreading in the environment, so both salmon food products and wild organisms become a source of resistant bacteria that can be transmitted to humans as foodborne contaminants (Lozano-Muñoz et al., 2021). Salmon culture farms played a role in the incidence of ARB in sediments, showing an important decrease in the number of ARB at greater distances from the farms (Buschmann et al., 2012).

There is a high-risk of antibiotic contamination in Chile, according to a recent review of ARB. Reverter et al. (2020) calculated the multi-antibiotic resistance index (MAR) of aquaculture-related bacteria (11.274 isolates) for 40 countries (representing for 93% of global animal aquaculture production) and Chile had a MAR index of 0.28, i.e. above 0.2, the threshold value considered as a high-risk antibiotic contamination index.

**Figure 6:**
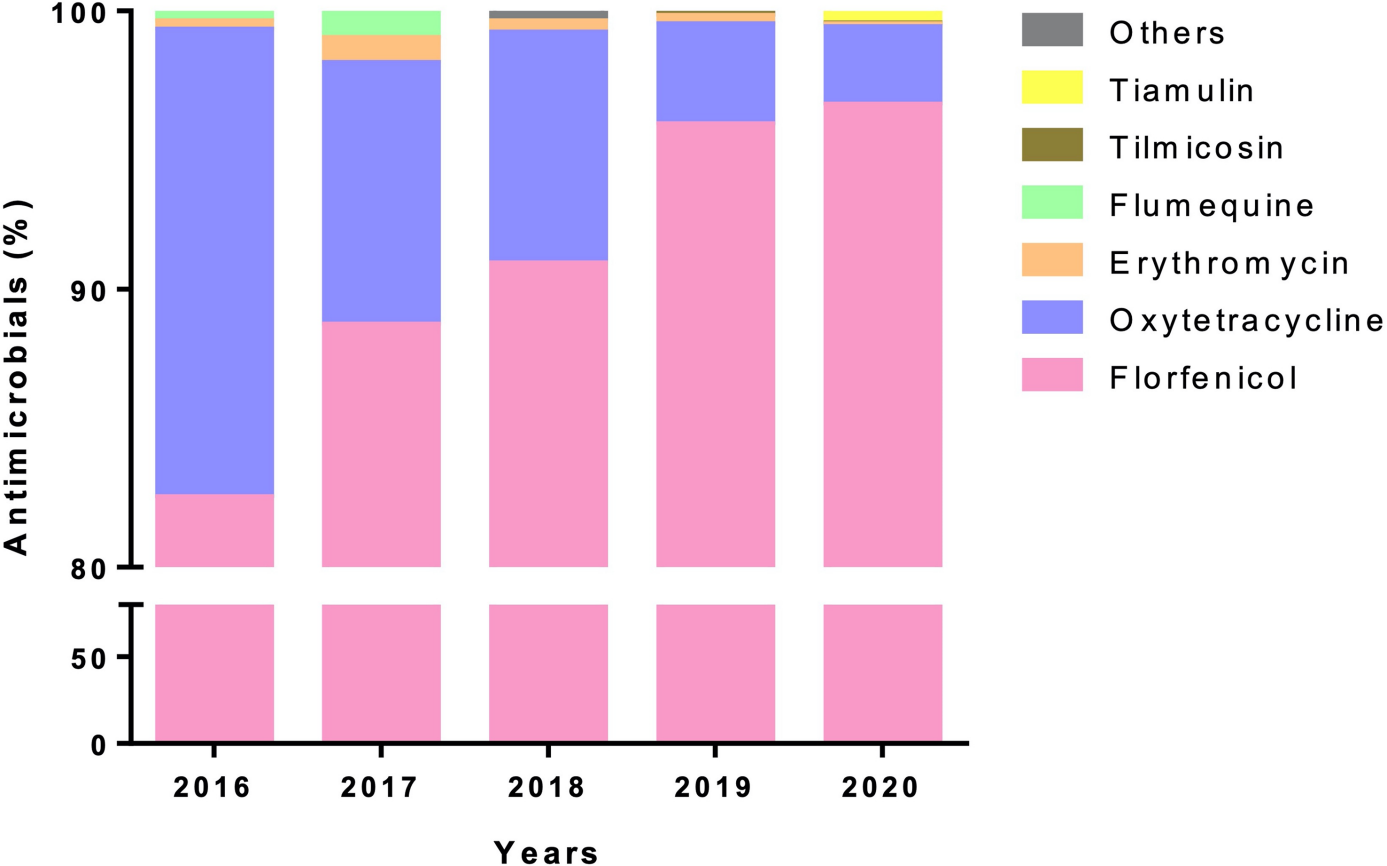
Use of antimicrobial by aquaculture industry in Los Lagos Region. Percentage of diverse antimicrobials (y-axis) used in Los Lagos Region during 2016-2020.

The main infectious disease facing the aquaculture industry in Chile is the Salmonid Rickettsial Syndrome (SRS). This disease is caused by the Gram-negative and intracellular bacterium *Piscirickettsia salmonis*, which mostly affects salmonid species in saltwater. On the other hand, in the freshwater phase, antibiotics are mostly directed against Flavobacteriosis and Renibacteriosis. Florfenicol and oxytetracycline represented for 98.7% and 1.25% of the total antimicrobials used in seawater, respectively (SERNAPESCA, 2020) (**Figure 6**). Despite the use of antibiotics and vaccines as key strategies to combat *P. salmonis*, all initiative has been unsuccessful (Flores-Kossack et al., 2020). Antibiotics have been used as a tool to maintain high production rates, resulting in overuse under conditions of high uncertainty and low effectiveness (Millanao et al., 2018; Jara et al., 2021).

The selection of resistant bacteria and the dissemination of ARGs to other bacteria in the host or ecosystem of cultivation areas has been discussed previously (Miranda et al., 2018; Ibrahim et al., 2020). In the last decade, Chilean companies showed a consistent increase in the number of antimicrobials used by salmonid farms, from 143.2 tons in 2010 to 379.6 tons in 2020 (97.51% in seawater and 2.49% in freshwater), which is equivalent to 4071.8 tons of antibiotics that were, for the most part, supplied through pelleted feed (**Figure 5A**) (OCEANA, 2020; SERNAPESCA, 2020). According to the Chilean Salmon Antibiotic Reduction Program (CSARP) 2020 report, Chilean farmed salmon had a use of active ingredient of antibiotic in the last five years of 382.5 tons in 2016, 393.9 tons in 2017, 322.7 tons in 2018, 334.1 tons in 2019, and 379.6 tons in 2020 (SERNAPESCA, 2020). Although its use has decreased compared to the amount used years ago, excessive amounts are still used. Compared to Norway, in 2018, Chile used 550 times more antibiotics than the industry in that country, the world’s leading producer of Atlantic salmon (OCEANA, 2020). Three years later, only 1.3% of salmonids (*Salmo salar* and *Oncorhynchus mykiss*) farming areas used antibacterial treatment, with 222 kg of antibiotics to produce 1.4 million tons of farmed fish. The significant decrease in the usage of antibacterial agents in Norwegian aquaculture from 1987, when it amounted to 48 tons or 876 mg/PCU (mg active substance/population correction unit) to 0.15 mg/PCU in 2019, it is mainly attributed to the introduction of effective vaccines against bacterial diseases in *Salmo salar* and *Oncorhynchus mykiss* but also to the prevention of bacterial diseases and their spread (Henriksson et al., 2018; NORM/NORM-VET, 2019). It is important to note that the maximum allowed dose of the antibiotic florfenicol in freshwater salmonid aquaculture is 10 to 15 mg per kg of fish, but Chile used around 49 times more than the regulated dose in 2017 (Lozano-Muñoz et al., 2021).

In farming areas, selection of resistant bacteria and dissemination of resistance elements to other bacteria has previously been described, both from the host and the environment (Miranda et al., 2018; Ibrahim et al., 2020). Unlike in Norway, where the state collaborates with companies to certify salmon farms by the Aquaculture Stewardship Council (ASC), in Chile fishery health policies are not well aligned with the ASC standards for salmon, and the country’s regulations do not prevent the use of antibiotics classified as critical to human health by the WHO (Luthman et al., 2019; Lozano-Muñoz et al., 2021).

The threat of AR must be urgently addressed through the implementing of national strategies in accordance with international standards that include both, the prudent use of antimicrobials on marine farms and investment towards a “One Health Concept” approach that combines human, animal, and environmental health (Lozano-Muñoz et al., 2021).

On the other hand, the increasing development of this industry in Chile, as well as the intensive use of antimicrobials, has not been accompanied by the scientific research necessary to understand the impact of the intensive use of antibiotics in this industry. Information on the ecological and environmental consequences of antibiotics use in fish farming is still scarce, with the presence of traces of antibiotics detected in sediments associated with salmon farming centers (Miranda et al., 2018), even in the marine environment up to 8 km from aquaculture sites, which could select for bacteria with multiple resistance in that environment (Millanao et al., 2018).

According to the Report of emissions and transference pollution issued by the Environment Ministry in 2019, 80% of the waters discharged in Los Lagos Region come largely from the aquaculture industry (52% mussel farming, 34% fish farming) while that 20% come from other industries (RETC, 2020). Therefore, although there is a lack of information on the interaction between salmon and mussel farming, thus, a competition for space and the use of water columns to establish concessions for the cultivation of *Mytilidae*, because both activities require the same environmental, oceanographic, and boundary conditions. Recently, several *Mytilus* spp. reared on different years and distance from salmon farms were analyzed to study their bacterial microbiota and susceptibility to florfenicol and oxytetracycline of their bacterial isolates (Ramírez et al., 2022). No antibiotic was detected on *Mytillus* samples, and *Mytilus* microbiota composition and minimum inhibitory concentration (MIC) values were associated with proximity to salmon farms, sampling years, and their interaction. However, author discussed that other study design are needed to confer causality. Importantly, bacterial genera of isolates with high MIC (≥ 64 μg mL^−1^) represented a low proportion of the microbiota identified with sequencing of the 16S rRNA gene. This urges the need to include more comprehensive methods, such as metagenomic to better describe the bacterial resistome, and HM resistance (Ramírez et al., 2022). In the aquaculture industry, antibiotics contained in feed or fish feces can diffuse into the sediments and be carried to distant sites by ocean currents, where they exert selective pressure and select ARB (Rasul and Majumdar, 2017). In addition, MIWTP plays a key role because they are considered the main “hot spot” of ARGs and spread bacteria in the environment because their wastewater discharges into several source waters like rivers, lakes, runoff, or groundwater end up reaching the oceans (Bitton, 2005). Finally, the risk that exists in the aquaculture areas of the Aysén and Los Lagos Regions is worrying, where the high concentrations of Zn present in seawater can mean an increase in resistance to antibiotics, with the consequent risk for human health. In 2010, Peltier and collaborates, reported that sub-toxic levels of Zn can increase the AR in *Escherichia coli* to tylosin, oxytetracycline and ciprofloxacin among culturable bacteria in wastewater treatment at low antibiotic levels, suggesting a cross-resistance associated from pre- and/or co-exposure to Zn (Peltier et al., 2010). This co-selection seems to be a consequence of the Zn exposure increase the abundance of MGEs, such as integrons and insertion sequences, with a significative association between ARGs and MGEs, suggesting that Zn enhance the potential for horizontal transfer of ARGs (Tongyi et al., 2020).

## Conclusion

**Figure 7:**
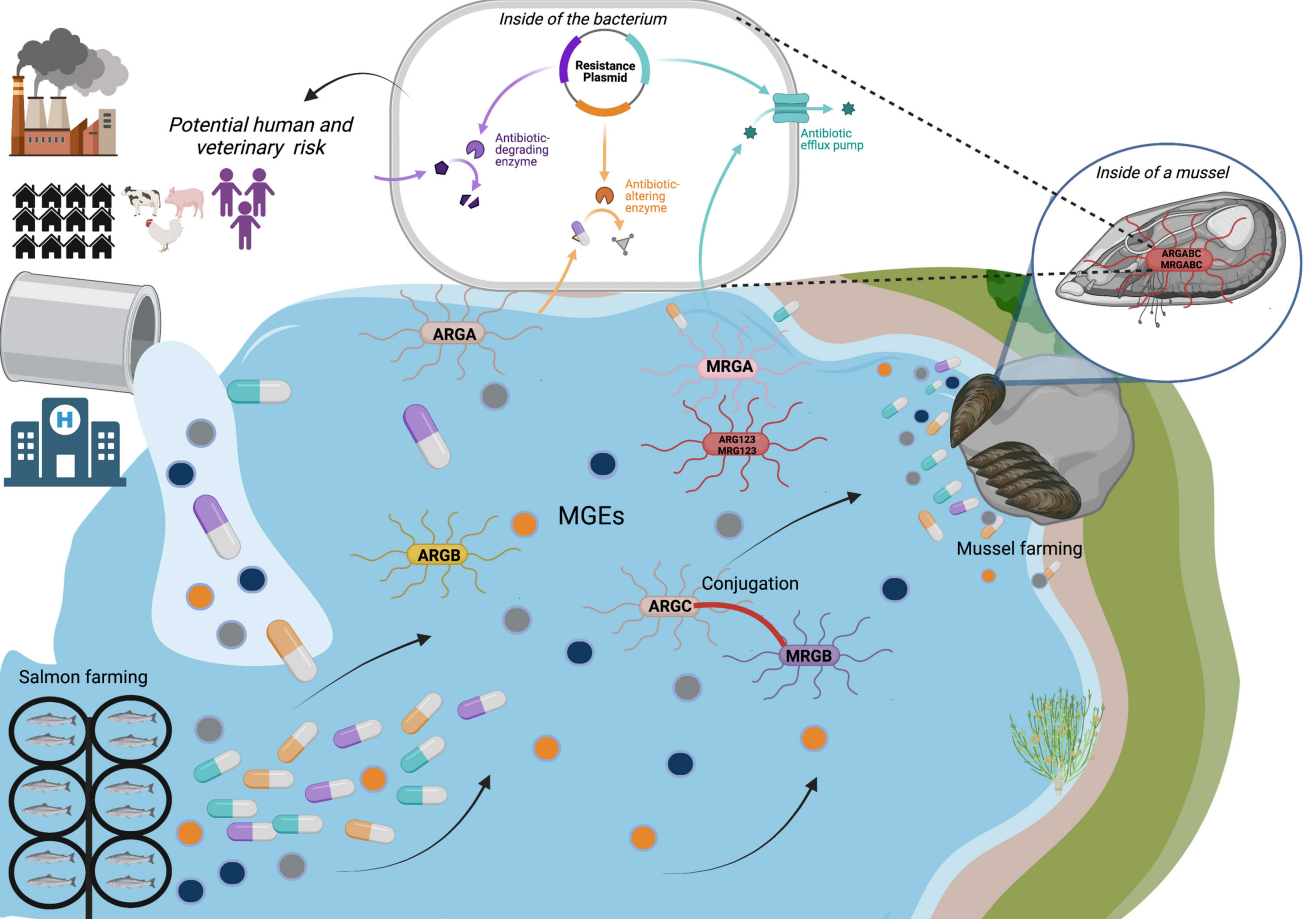
Possible risk of mussel’s cultivation in Los Lagos Region. Discharge of antibiotics and heavy metals from diverse industries in Los Lagos Region implies a possible risk to humans and animals, which consume mollusks raised in these areas. The presence of antibiotics and heavy metals could favor co-selection, co-resistance and/or cross-resistance mechanisms between bacteria with mobile genetics elements (MGEs) and different antimicrobial resistance genes (ARGA, ARGB, ARGC) or different metal resistance genes (MRGA, MRGB, MRGC) inside the bacterial components of the mussels’ microbiota, rising the risk of antimicrobial-resistance genes transfer to consumers.

The co-occurrence of resistance to HMs and antibiotics has a profound impact on health and modern antibiotics use, considering that selection pressure for HMs may favor the proliferation of antibiotics resistance *via* co-selection of ARGs and HMRGs, given the high potential for co-transfer of both types of resistance (Li et al., 2017). Since the last decade, the increase of ARB due to mechanisms such as cross-resistance to HMs and the co-regulation of the pathways that determine resistance in these bacteria has been credited (Berg et al., 2005; Akinbowale et al., 2007a; Akinbowale et al., 2007b; Wales and Davies, 2015). Consequently, there is a high probability that these HMs are taken up by marine bacteria. In Los Lagos Region, the presence of antibiotics and HMs as water pollutants, coming from the intensive activity of salmonid farming, as well as from the discharge of sanitary water for domestic use, or from hospitals, health centers, and industrial discharges (**Figure 7**), denotes a danger to human, due to the possible HGT between antimicrobial-resistant marine bacteria and human pathogens. The questions that arise are, how would these genes be affected in areas highly contaminated with Zn?, which is one of the HMs most associated with AR? Are human pathogenic species of *Vibrio* that are part of the microbiota of *Mytilus chilensis* cultivated in Los Lagos Region, carriers of multiple resistance genes? Could they mean a risk for human consumers? Therefore, it is of utmost importance to establish a surveillance plan for the occurrence of bacterial resistance phenotypes, which should be extensively investigated to provide a real reference of the potential for co-selection in the genotype. It is necessary to identify all potential co-selection agents and their roles in the dissemination of AR in the human-associated environment (Li et al., 2017), which will contribute to risk assessment of AR under current clinical/environmental management.

## Author Contributions

AP, LP, and KG conceived the idea. DR and CL-J wrote about heavy metals contamination. VJ and CI wrote the paragraphs of AR and bivalve immunity. CM, PN, and AP develop the paragraph of co-resistance’s mechanisms. KG, DR, and SR-C wrote about the Chilean situation. All authors read, discussed, and approved this manuscript.

## Funding

This study was funded by competitive funds of Universidad de Las Américas #PI202019.

## Conflict of Interest

The authors declare that the research was conducted in the absence of any commercial or financial relationships that could be construed as a potential conflict of interest.

## Publisher’s Note

All claims expressed in this article are solely those of the authors and do not necessarily represent those of their affiliated organizations, or those of the publisher, the editors and the reviewers. Any product that may be evaluated in this article, or claim that may be made by its manufacturer, is not guaranteed or endorsed by the publisher.
